# Comprehensive Transcriptome Meta-analysis to Characterize Host Immune Responses in Helminth Infections

**DOI:** 10.1371/journal.pntd.0004624

**Published:** 2016-04-08

**Authors:** Guangyan Zhou, Mary M. Stevenson, Timothy G. Geary, Jianguo Xia

**Affiliations:** 1 Institute of Parasitology, McGill University, Sainte Anne de Bellevue, Quebec, Canada; 2 Centre for Host-Parasite Interactions, McGill University, Sainte Anne de Bellevue, Quebec, Canada; 3 Departments of Medicine and Microbiology and Immunology, McGill University, Montreal, Quebec, Canada; 4 Department of Animal Science, McGill University, Sainte Anne de Bellevue, Quebec, Canada; Leiden University Medical Center, NETHERLANDS

## Abstract

Helminth infections affect more than a third of the world’s population. Despite very broad phylogenetic differences among helminth parasite species, a systemic Th2 host immune response is typically associated with long-term helminth infections, also known as the “helminth effect”. Many investigations have been carried out to study host gene expression profiles during helminth infections. The objective of this study is to determine if there is a common transcriptomic signature characteristic of the helminth effect across multiple helminth species and tissue types. To this end, we performed a comprehensive meta-analysis of publicly available gene expression datasets. After data processing and adjusting for study-specific effects, we identified ~700 differentially expressed genes that are changed consistently during helminth infections. Functional enrichment analyses indicate that upregulated genes are predominantly involved in various immune functions, including immunomodulation, immune signaling, inflammation, pathogen recognition and antigen presentation. Down-regulated genes are mainly involved in metabolic process, with only a few of them are involved in immune regulation. This common immune gene signature confirms previous observations and indicates that the helminth effect is robust across different parasite species as well as host tissue types. To the best of our knowledge, this study is the first comprehensive meta-analysis of host transcriptome profiles during helminth infections.

## Author Summary

Many studies have been conducted to understand the immune modulatory effects in helminth infections. To determine whether there is a common transcriptomic signature characteristic of the helminth effect, we performed a comprehensive meta-analysis of publicly available gene expression datasets. The results revealed a distinct pattern of gene expression that is consistent across multiple helminth species and host tissue types, with upregulated genes dominated by those involved in immune regulation, Th2 immunity and inflammatory responses.

## Introduction

Helminth infections, also known as helminthiases, are estimated to affect > 2 billion people and especially prevalent in developing countries. Despite recent progress in helminth control programs, much work remains to be done to achieve eradication [1]. To develop effective intervention strategies, it is critical to understand in detail the molecular mechanisms underlying host immune responses to these parasites.

The ability of helminths to manipulate the host immune system allows the infection to persist for years without being eliminated. This helminth effect has been observed with vastly divergent parasites, including nematodes, trematodes and cestodes, and has attracted increasing attention over the past decade. Although the term “helminth” is used to describe a diverse array of phylogenetically different organisms, evolutionary pressure from the host immune system is likely to have selected for convergent phenotypes among these pathogens with immuno-modulatory capacity [2, 3]. Indeed, host immune responses to helminthiases are typically characterized as Th2-type accompanied by general immune down-regulation [4]. Th2 responses involve the cytokines interleukin (IL)-3, IL-4, IL-5, IL-9, and IL-13 and additional features such as eosinophilia, goblet cell and mast cell hyperplasia, as well as alternatively activated macrophages [5]. Immunosuppression, on the other hand, is mainly mediated by regulatory T cells secreting IL-10 and TGF-beta.

Helminth-mediated immunomodulation is an active process that requires live parasites and can be carried out through different mechanisms [4]. In particular, helminth parasites secrete soluble mediators that can interact with host immune cells through evolutionarily conserved chemical dialogs [6]. For instance, studies have shown that a schistosome cysteine protease induces IgE production [7] and proteins secreted from adult *Nippostrongylus brasiliensis* elicit strong Th2 immune responses [8]. Host immunity can be modulated at different life-cycle stages of helminths, including larvae and eggs [9, 10]. Additionally, immunomodulation can be mediated through direct interactions between helminth surface molecules and host cells. For example, glycoconjugates and lipoproteins expressed on the surface of helminths can interact with host cells and shape immune responses [11]. Molecular mimicry is also utilized by these parasites to alter the host immune response. Homologues of anti-inflammatory molecules such as TGF-beta [12], macrophage migration inhibitory factors [13] and SOCS-1 [14] are produced by helminths. Finally, recent studies have shown that the helminth effect may also be mediated indirectly via changes in the gut microbiota [15, 16].

Host immune responses during helminth infections represent the outcome of eons of co-evolution between parasites and hosts. In addition to the immunomodulation mediated by helminths to prolong the infection, hosts have also evolved mechanisms to limit the damage associated with helminth infections. Host responses to helminths are characterized by a trade-off between immunity against the parasite and immunopathology caused by prolonged inflammatory responses. Indeed, the host often favors tolerance of helminth infection over complete parasite destruction to limit immune-mediated damages [5]. Studies in mouse models have shown that IL-4 deficient mice infected by schistosomes die due to excessive inflammation eight weeks post-infection, while wild-type mice enter a chronic phase of schistosomiasis [17]. Maintaining a regulated immune response through Tregs or a modified Th2 immune response is pivotal for the well-being of the host. Upon immune dysregulation, pathologies can arise as in the case of hepatic fibrosis in schistosomiasis or elephantiasis associated with lymphatic filariasis [3].

Understanding the helminth effect will not only provide enlightenment on fundamental aspects of helminthiases, but can also serve as a basis to develop new treatment strategies for immune disorders, such as asthma, allergy, inflammatory bowel disease and type 1 diabetes. Helminthiases are thought to help prevent the onset and reduce the severity of these diseases, based on observations that higher prevalence of parasitic infections in a population is associated with lower incidence of immune-related diseases [18]. Subsequent experimentations on non-obese diabetic mice infected with helminths revealed that they are able to prevent the onset of type 1 diabetes [19, 20]. Many human studies have explored the therapeutic value of helminths to treat and prevent immune-mediated diseases, particularly with nematodes. Experimental data have shown positive results in the treatment of inflammatory bowel disease as well as multiple sclerosis [21]. The immunoregulatory environment created by helminth infections extends beyond reduction of responsiveness to parasite antigens [22], as the infected patients are also less responsive to other antigens such as vaccines, allergens and self-antigens [23].

Global gene expression profiling using microarray or RNAseq technologies has been widely used to reveal biological and cellular processes involved in host immune responses during infection. The growing number of available datasets in public repositories such as Gene Expression Omnibus (GEO) [24] and ArrayExpress [25] offers excellent opportunities to perform comprehensive analyses by integrating multiple studies to identify robust gene expression signatures that would be otherwise unidentifiable in individual studies [26]. The benefits of a meta-analysis lie in the ability to limit or eliminate potential biases associated with individual studies, and to improve statistical power to enable detection of subtle but biologically meaningful variations through increased sample sizes. It has been widely used to identify patterns of gene expression in complex pathophysiological conditions, including infection and inflammation [27, 28], aging [29] and cancer [30]. For instance, in a landmark meta-analysis study by Jenner and Young, gene expression datasets from both *in vitro* and *in vivo* infection models were combined to reveal a common host transcriptional response against a wide array of different pathogens (bacteria, virus and protozoa) across different cell types [27]. To the best of our knowledge, no comprehensive meta-analysis of helminthiases has been reported. The hypothesis of our current study is that the helminth effect can be identified and characterized as a unique gene expression signature across different parasite species and host tissues, and by applying suitable meta-analysis procedures to integrate different datasets, this signature can be revealed to provide insights into the molecular mechanisms underlying the interactions between host and helminth parasites.

## Materials and Methods

### Data collection

**Fig 1:**
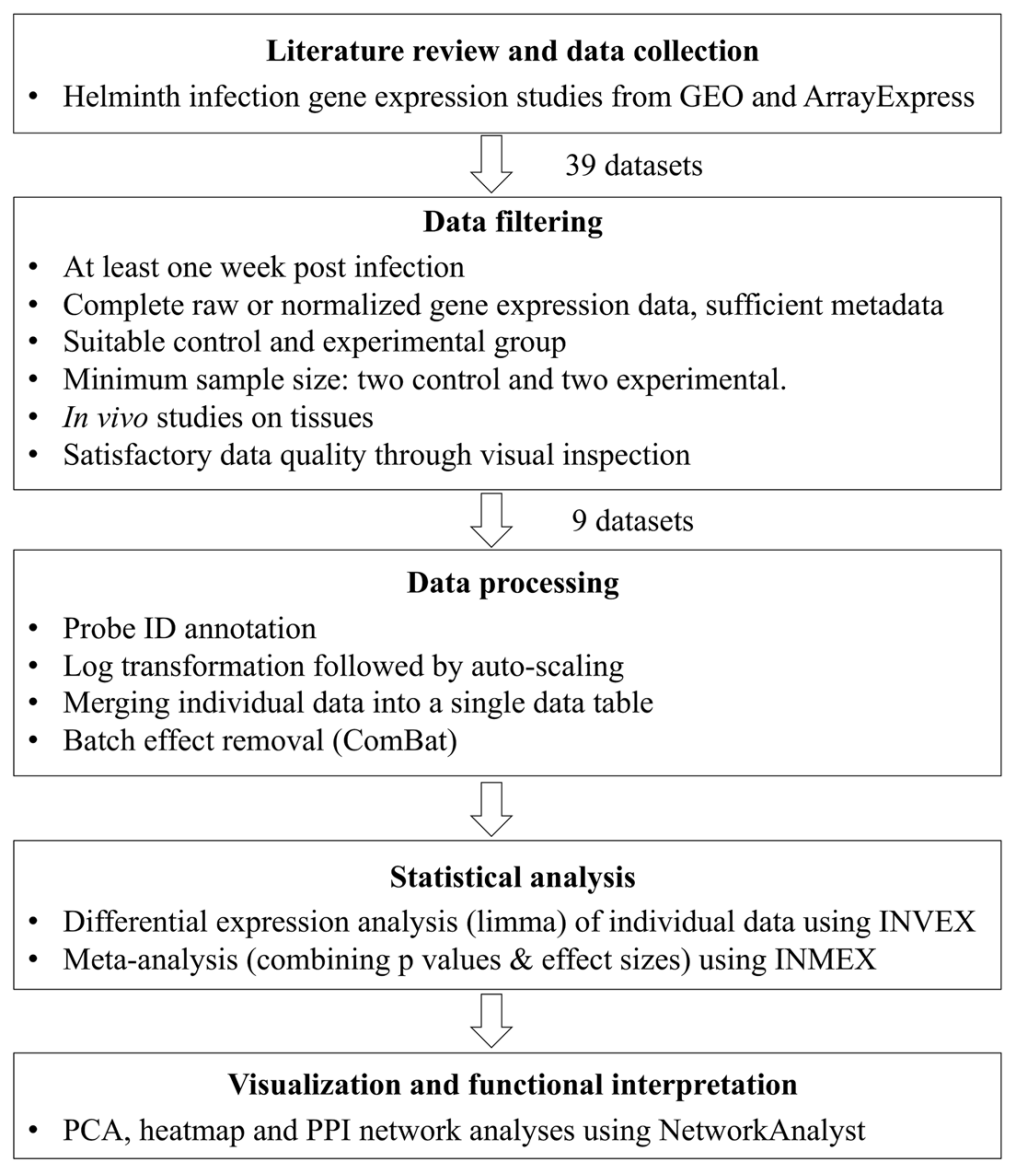
The meta-analysis flowchart. The process consists of five main stages. The main tasks in each stage are described in the corresponding box.

GEO and ArrayExpress were queried during June and August 2015 to collect datasets related to helminthiases. We focused on mouse models because our initial survey indicated that the majority of available datasets are based on this host. The following criteria were applied during data selection: (1) immune response profiling after at least one week post-infection; (2) complete raw or normalized gene expression data available; (3) presence of suitable control group; (4) minimum of four samples with two controls and two cases; and (5) *in vivo* studies with tissue samples from wild-type mice. As our focus is on normal immune responses, we excluded *in vitro* studies of particular cell types, experiments using transgenic hosts or experiments focusing on specific research subjects that may result in transcriptional changes not representative of normal infected states (i.e., study of gene expression after drug treatment). Several additional datasets were excluded due to lack of metadata or poor quality. The detailed procedures are described in **Fig 1**.

### Data processing and differential expression analysis of individual datasets

Individual data annotation and analysis were performed using the INVEX web-based tool [31]. In particular, probe IDs from different microarray platforms were converted to Entrez gene IDs. If more than one probe was mapped to the same gene, then the mean gene expression value of these probes was used for that gene. After probe annotation, individual datasets were log transformed followed by auto scaling. We then performed differential expression (DE) analysis between the control and the infected groups for individual datasets using the limma approach [32]. The resulting gene expression patterns were visually inspected using the interactive heatmap.

### Batch effect adjustment

To reduce potential study-specific batch effects, the pre-processed and normalized data sets were further subjected to the well-established ComBat procedures [33]. The method has been specially designed for adjusting batch effects in microarray expression data with small sample sizes. Firstly, genes absent in more than 80% of samples are eliminated and the remaining ones are standardized to have similar overall mean and variance; Secondly, information is pooled across genes from a batch to estimate batch effects that affect many genes such as increased level of expression and higher variability; Thirdly, these batch effects are then adjusted to obtain normalized data. To compare the sample clustering patterns before and after applying the ComBat procedures, the results were visually examined using the principal component analysis (PCA). The first two principal components were then used to create a scatter plot with the colors and shapes of the data points corresponding to different studies or tissue types for visual assessment.

### Statistical meta-analysis

The meta-analysis was performed using the web-based tool—INMEX [34]. Two popular meta-analysis methods were explored–the p-value combination using Fisher’s approach, and the effect size combination using the random effect model. Effect size is a standardized difference defined as the difference between group means divided by its standard deviation (i.e. Z-score). In particular, Fisher’s approach combines the p values from several independent tests bearing upon the same overall hypothesis and evaluates the significance using chi-squared tests; while effect size combination takes into consideration both the direction and magnitude of gene expression changes to generate more biologically consistent results. The random effect model was chosen because there were significant cross-study heterogeneities based on the Cochrans’ Q test. An adjusted p value of 0.01, based on the false discovery rate using the Benjamini–Hochberg procedure, was used to select DE genes.

### Data visualization and functional interpretation

The merged expression data containing all DE genes was subjected to complete hierarchal clustering and explored using various interactive visualization methods offered within NetworkAnalyst [35]. Genes within the identified major clusters were subjected to over-representation analyses using Gene Ontology [36], PANTHER classification system [37], and Reactome pathways [38]. To characterize the relationships among those co-upregulated genes, we projected them into the mouse protein-protein interaction (PPI) network based on InnateDB [39]. Two subnetworks were further created for up-regulated and down-regulated clusters, respectively, for visual inspection and functional assessment.

## Results

### Data collection and filtering

As shown in **Fig 1,** after data filtering based on the inclusion-exclusion criteria, nine studies were left, involving five different helminth parasites (*Schistosoma mansoni*, *S*. *japonicum*, *Fasciola hepatica*, *Trichinella spiralis* and *Nippostrongylus brasiliensis*) collected from four different tissues (lung, intestine, liver and diaphragm) and three mouse strains (BALB/c, C57BL/6 and CBA), with a total of 55 samples [40–47]. The details of the nine datasets are provided in **Table 1**. These pathogens represent two divergent trematode groups and two divergent nematode clades. The complete list of the 39 datasets together with detailed annotation is given in **S1 Table**.

**Table 1:** The nine public gene expression datasets included in our meta-analysis.

Datasets	Platforms	Tissues	Parasites	References
E-GEOD-25713	Illumina MouseWG-6 v2.0	Liver	*S*. *japonicum*	[42]
GSE41941	Illumina MouseRef-8 v2.0	Liver	*S*. *japonicum*	[41]
GSE69588	Affymetrix Mouse Gene 1.0 ST Array	Liver	*F*. *hepatica*	[45]
E-GEOD-59276	Illumina MouseRef-8 v2.0	Liver	*S*. *japonicum*	N/A
GSE48936	Affymetrix Mouse Gene 1.0 ST Array	Lung	*S*. *mansoni*	[46]
E-GEOD-3414	Affymetrix Mouse Genome 430 2.0	Lung	*N*. *brasiliensis*	[43]
E-GEOD-5555	Affymetrix Mouse Genome 430 2.0	Lung	*N*. *brasiliensis*	[44]
GSE67136	Illumina MouseRef-8 v2.0	Diaphragm	*T*. *spiralis*	[47]
E-GEOD-31265	Agilent Mouse 44K custom	Intestine	*S*. *mansoni*	[40]

### Processing and differential expression analysis of individual datasets

After probe annotation and filtering, we identified a total of 11,573 genes that are common across all nine datasets. Using an adjusted p-value cut-off of 0.05, an average of 9.06% of genes was found to be significant. Most of the results are similar to those reported in the original publications. In some cases, however, we notice that different data normalization procedures could significantly affect the number of DE genes identified.

### Batch effect adjustment

**Fig 2:**
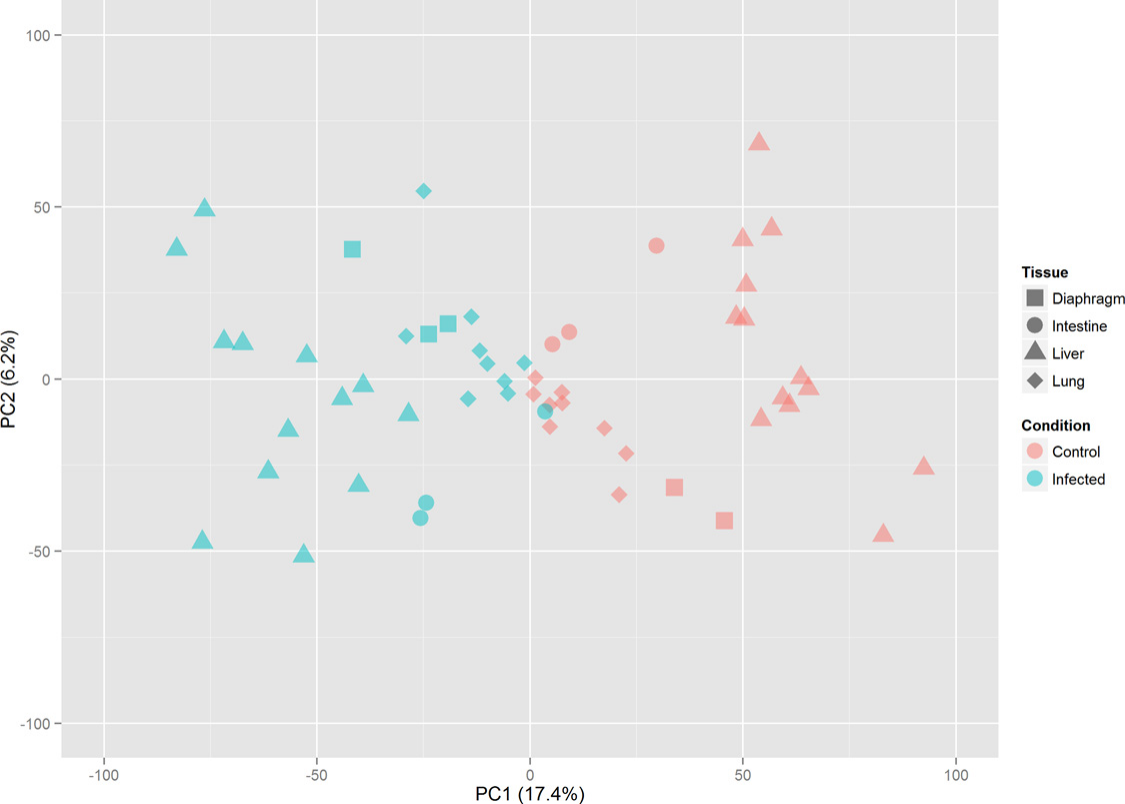
Principal component analysis of the combined dataset after batch effect correction. All samples are represented by different symbols with shapes according to their tissue origins and colors based on their experimental conditions.

As our goal is to identify the core immune response signature across different parasite species and tissue types, it is important to estimate the effect of these factors before meta-analysis. As shown in **S1 Fig**, the 55 samples are tightly clustered mainly according to the original studies. After batch effect correction using the ComBat approach, this study-specific clustering pattern mainly disappeared (**S2 Fig**) and the resulting PCA plot showed clustering based primarily on control and infection groups on the first PC (**Fig 2**). Another consideration is tissue-specific effects. These effects are hard to assess due to the paucity of available studies—both intestine and diaphragm are only represented by a single study each. However, the PCA plot (**Fig 2**) clearly indicates that the overall clustering patterns are dominated by the helminth effect. We further explored the corresponding PCA loading plots and confirmed that the main genes driving the separation between control and infection groups are immune-related rather than tissue-related. We also investigated the possible effects of different mouse strains. As shown in **S3 Fig**, no particular clustering patterns were detected for different strains, indicating that the strain effects are unlikely to play a significant role in our meta-analysis focusing on helminth effect. **S4 and S5 Figs** further show the changes of two individual genes across different datasets before and after applying the ComBat procedures to help illustrate the batch effect correction procedures.

### Statistical meta-analysis

**Fig 3:**
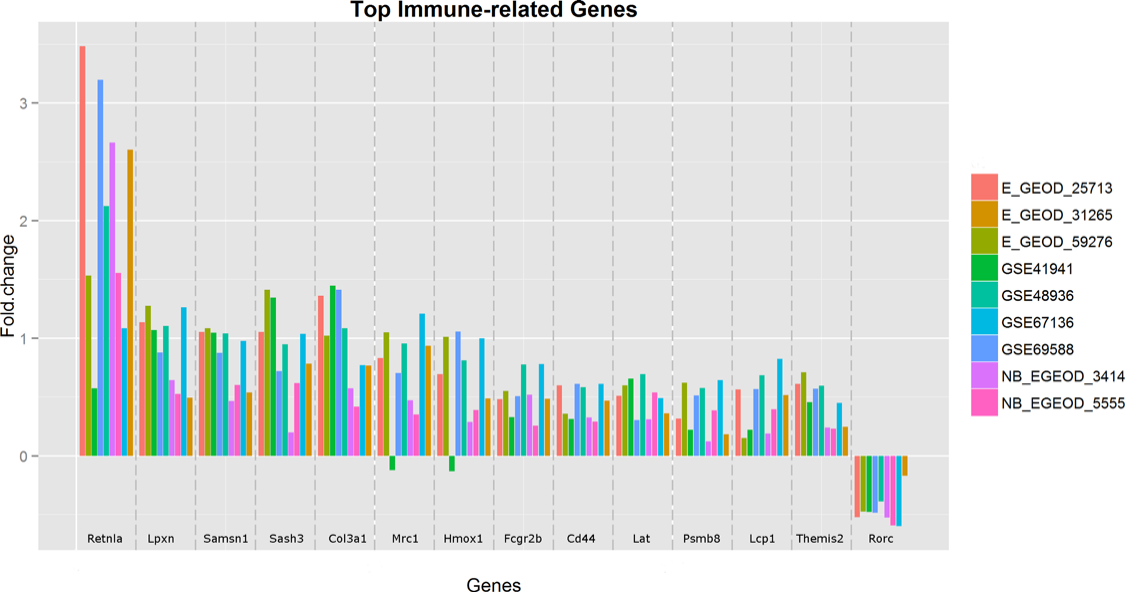
The fold changes of top 14 immune genes across different datasets. The small negative fold changes of *Mrc1* and *Hmox1* genes in the dataset GSE41941 (containing two replicates) are likely due to study-specific bias.

**Fig 4:**
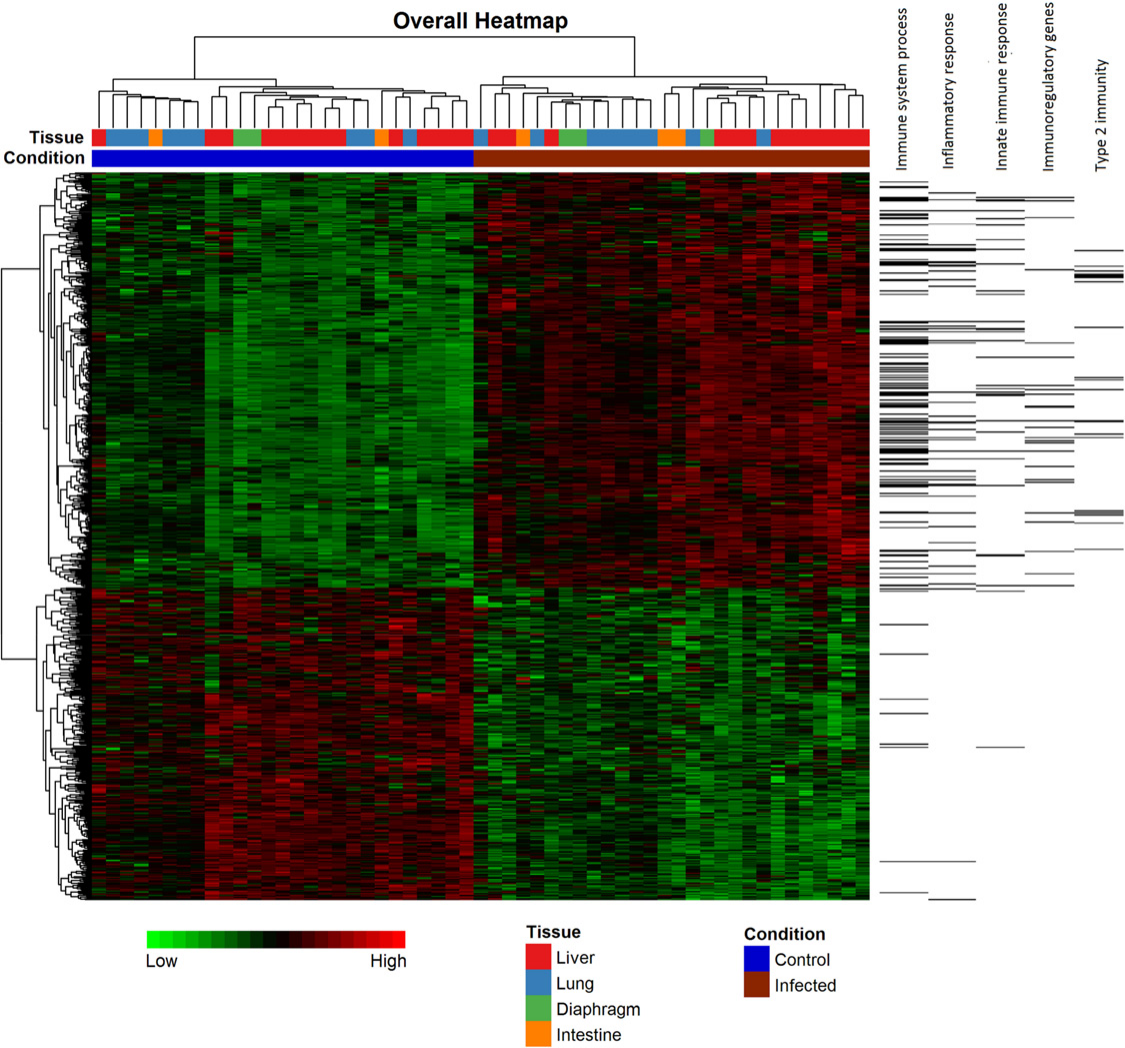
The heatmap of DE genes identified during meta-analysis. A total of 691 DE genes were identified (394 up-regulated and 297 down-regulated). The horizontal lines on the right indicate those genes belong to several immune related functional groups.

Over 5,000 DE genes were identified with Fisher’s approach using an adjusted p value cut-off of 0.01. However, close inspection indicated a significant proportion of genes showing contradictory expression changes in different studies, which may be caused by the inefficiency of Fisher’s approach when very small numbers of samples are available in individual datasets. In contrast, combining effect sizes using the random effect model produced more biologically meaningful results. Using an adjusted p-value cut-off value of 0.01, we obtained 691 DE genes (**S2 Table**). The result table for all 11,573 genes is provided in **S3 Table.** The fold changes of the top 14 immune genes across all nine studies are depicted in **Fig 3**. It illustrates the strength of meta-analysis–improved statistical power for identification of consistent expression changes across different studies, and using the collective power to overcome potential bias in particular studies. For instance, the small negative fold changes of *Mrc1* and *Hmox1* genes in the *GSE41941* dataset (containing only two replicates) are most likely due to study-specific bias when compared to the overall larger positive fold changes in other datasets. The hierarchical heatmap (**Fig 4**) shows that these DE genes can be clustered into two distinctive groups, with one major group of 394 genes that are consistently up-regulated during helminth infection, and the other group of 297 consistently down-regulated genes. Among these 691 DE genes, 145 of them were uniquely identified only in the meta-analysis.

### Functional enrichment analysis

To gain insights into the main functions of those genes within the two major clusters, we performed functional enrichment analyses. PANTHER GO biological process enrichment analysis of all 691 genes showed that 133 genes are involved in “immune system process” (p-value of 2.32E-9). Among them, 123 are up regulated. Comparing with the genes responsible for common host responses against diverse pathogens identified by Jenner and Young [27], there is an overlap of 33 genes. The most significant overlap is found in chemokine and cytokine genes, followed by immune signalling genes. Among these genes, there are several sets of genes involved in immunological process that are overrepresented. Up-regulation of immune regulatory genes is particularly significant, with 27 genes identified as having inhibitory activity on immunological processes, such as negative regulation of B cell receptor signaling pathway (GO:0050859), negative regulation of antigen receptor-mediated signaling pathway (GO:0050858), negative regulation of interferon-gamma production (GO:0032689), *etc*. As for down-regulated genes, the vast majority of GO categories are related to metabolism.

Further inspection of the list of up-regulated genes shows that many of them are involved in Th2- related immune responses, including the Th2 cytokines *Il4* and *Il33* and others (*Ccl7* and *Jak3*). *Il4* can antagonize *Il1* activity by up-regulating the expression of *Il1r2*, a decoy receptor for *Il1* [48]. Interestingly, we observed that both *Il1rn*, an antagonist of *Il1*, and *Il1r2* are overexpressed; both genes encode proteins that inhibit activities of *Il1*. In addition, the list also contains many markers of alternatively activated macrophages (AAM) (*Mrc1*, *Retnla*, *Chi3l3* and *Trem2*) [49]. Unlike classically activated macrophages, AAM are induced by *Il4* and *Il13* produced by CD4^+^ Th2 cells and other cells during type 2 immune responses; AAM are characterized by the production and release of anti-inflammatory factors along with the ability to promote wound repair and extracellular matrix remodelling [49]. These main immune functional groups are discussed in detail below.

Enrichment analyses showed that 56% of the down-regulated genes are involved in metabolic processes (GO:0008152). Out of 47 GO annotations, 36 of them directly relate to metabolic process. The most significant down-regulated gene is glutathione *S-transferase alpha 3* or *Gsta3*, with a p-value of 5.14E-12 and combined effect size of -4.85. This gene is involved primarily in glutathione metabolism and also plays an accessory role in autophagy [50]. Interestingly, this gene is also found to be among the top up-regulated genes in mice model of type two diabetes [51]. Another down-regulated immune gene is *Rorc*. This gene is associated with induction of Th17 responses [52]. The main metabolic pathways affected are metabolism of lipids and lipoproteins. The group biological oxidation is also significantly down regulated. Down-regulation is also observed in four genes in the cytochrome P450 family. This family encompasses a large number of genes which play important roles in hormone synthesis and breakdown, cholesterol synthesis, vitamin D metabolism, and metabolism of xenobiotic compounds and bilirubin in the liver [53]. Overall, the magnitude of changes in expression changes is much smaller and more heterogeneous in these down-regulated genes than in genes in the up-regulated cluster. Indeed, using effect size as a reference for the magnitude of changes in gene expression, only 30% of down-regulated genes had an effect size ≥ 2 compared to 74% of up-regulated genes.

#### Cytokines and cytokines

The most significantly up-regulated functional group consists of genes encoding chemokines and cytokines. Chemokines are responsible for the recruitment of immune cells to the site of infection, while cytokines play important roles in regulating the immune system [54, 55]. Among the chemokines are the Ccl-chemokines (*Ccl2*, *Ccl3*, *Ccl4* and *Ccl7*) and Cxcl-chemokines (*Cxcl2*, *Pf4/Cxcl4* and *Cxcl9*). Other cytokine genes including *Osm*, *C4b*, *Il1rn*, *Ptn*, *Mdk a*nd *Tnfsf14* cytokine receptors are also significantly up regulated. This functional class includes *Cd2*, *Cd274*, *Cd300a*, *Cd3*3, *Cd3g*, *Cd40*, *Cd84*, *Clec10a*, *Clec4b1*, *Clec4d*, *Ifnar2*, *Il10ra*, *Il1r2*, *Ly9*, *Osmr*, *Slamf9*, *Tnfrsf11a*, *Tnfrsf11*b, *Tnfrsf4* and *Vcam1*, in addition to chemokine receptors *Ccr*5 and *Cxcr3*. Overexpression of these functional classes is commonly observed in infection-related gene expression studies, indicating their fundamental functions in host responses against diverse pathogens.

#### Pathogen recognition and initiation of immune responses

In addition to cytokines, many genes involved in pathogen-associated molecular pattern (PAMPs) recognition are up regulated. These genes play pivotal roles in host responses by recognizing molecular structures specific to pathogens and initiating appropriate innate immune responses [56]. They include genes involved in Toll-like receptor signalling such as *Cd14*, *Tlr7*, *Lgmn* and *Myd88*, as well as C-type lectin receptor genes such as *Clec7a*, *Clec4n*, *Clec4d* and *Clec10a* [57]. Additionally, MHC II genes, including *H2-Aa*, *H2-DMa* and *H2-Eb1*, were also upregulated. They are responsible for the presentation of exogenous antigens to naïve CD4^+^ T cells, leading to polarization to discrete subsets of Th cells [58]. Up-regulation of FCGR receptor genes, including *Fcgr1*, *Fcgr2b*, *Fcgr3* and *Fcgr4*, is also observed. They play important roles in both antigen presentation and immune regulation [59].

#### Immune signaling pathways

Another group of DE genes identified in our meta-analysis are involved in signal transduction. Genes in this category include those involved in interferon signalling: *CD44*, *H2-Aa*, *H2-Eb1*, *Irf5*, *Irf8*, *Fcgr1*, *Oas2* and *Ifnar2*. Additionally, the Dap12 signalling is affected. *Tyrobp* (DAP-12 adapter) and its related receptors *Trem2* and *Clec5a* are consistently up-regulated, while signaling genes have a mixed response in terms of association with infection, with about half of these genes being up-regulated. Affected PD-1 signaling genes include *H2-Aa*, *H2-Eb1*, *Lck*, *Cd3g*, *Cd274* and *Ptpn6*. Among the pathways presented above, interferon signaling showed the most pronounced up-regulation. These pathways are interconnected and play critical roles in immune responses. Most of these genes mediate immune activation, but a few are inhibitory genes. These inhibitory genes serve to maintain immune homeostasis, preventing excessive immune responses that may cause immunopathology.

#### Other immune response genes

We also detected DE genes in several smaller immune-related functional groups. These include inflammatory markers (*Lcn2* and *Saa3*), genes involved in reactive oxygen species generation (*Gpx7*, *Ncf1* and *Ncf4*), leukotriene production (*Alox5ap*), 25-hydroxycholesterol production (*Ch25h*), production of histamine (*Hdc*), and EGF-like module containing mucin-like hormone receptor-like 1 (*Emr1*). These genes indicate an ongoing inflammatory response and activation of innate defense mechanisms against helminths, although not necessarily effective in eliminating the pathogens.

#### Other functional categories

Aside from immune system processes, Reactome pathway analysis showed that hemostasis is affected, with 39 DE genes related to this function and 33 of which are up-regulated. Changes in the expression of these genes are most likely a consequence of tissue damage caused by parasite infection. Other genes mediating tissue remodeling are also up-regulated, including genes encoding proteins in the matrix metalloproteinase family (*Mmp2*), collagens (*Col3a1*, *Col5a1*, *Col6a1*, *Col6a2*, *Col14a1* and *Col15a1*) and tissue inhibitor of metallopeptidase 1 (*Timp1*).

### Top immune genes

The complete DE gene list is provided in **S1 Table**. To further characterize our data, we examined the top 50 DE genes (**Table 2**). Among them, 39 are up-regulated and 11 are down-regulated. The “Immune effector process” category is highly represented among these top genes. This category includes *Col3a1*, *Fcgr2b*, *Cd44*, *Lat*, *Sash3*, *Rorc*, *Hmox1*, *Lcp1*, *Anxa1*, *Lpxn*, *Psmb8*, *Themis2* and *Samsn1*. *Cd44* is a receptor involved in cell-cell and cell-matrix interactions that has diverse roles including maintaining tissue integrity, mediating lymphocyte activation, homing and interacting with diverse chemical factors and hormones [60]; *Lat* is involved in the mediation of T-cell signaling [61]; *Sash3* plays an important role in the differentiation of T cells [62]; *Psmb8* is a subunit of the immunoproteasome whose function notably includes the processing of antigens [63]. Interestingly, among these DE genes, only *Rorc* is down-regulated. This gene is associated with the pro-inflammatory phenotype and induction of Th17 responses, a type of immunity that has been associated with autoimmune diseases [52]. A previous study reported immunosuppression of Th17 response mediated by helminth infection resulted in attenuation of autoimmune disease [64]. It is interesting to note that *Fcgr2b*, *Col3a1*, *Hmox1* and *Samsn1* function in immunosuppression (GO:0050777). Additionally, *Lpxn* is involved in negative regulation of B-cell signaling [65] and *Anxa1* participates in the glucocorticoid-mediated anti-inflammatory effects [66]. *Ch25h* is not classified as an immune-related gene, but is associated with activation of macrophages and dendritic cells by various TLR ligands [67]. It catalyzes formation of 25-hydroxycholesterol [68], a compound that has wide array of regulatory effects on immune cells. *Emr1* and *Themis2 a*re involved in macrophage activation [69, 70], while *Mrc1* and *Retnla* over-expression suggests the presence of AAM, which are associated with Th2-type immune responses [49]. Additionally, the overexpression of *Hdc*, a gene in the histamine synthesis pathway [71], suggests that activation of mast cells is associated with helminth infection, as noted previously [72]. All these changes provide evidence to support a helminth infection-related signature of Th2-type immune response.

**Table 2:** The top 50 DE genes. The genes are ranked by their effect sizes and separated based on their directions of changes.

Gene Symbol	Gene Name	Effect Size	P-value
Retnla	resistin like alpha	5.66	1.25E-06
Lpxn	leupaxin	5.16	4.77E-06
Fermt3	fermitin family homolog 3 (Drosophila)	4.94	4.68E-07
Ms4a7	membrane-spanning 4-domains, subfamily A, member 7	4.89	1.57E-08
Samsn1	SAM domain, SH3 domain and nuclear localization signals, 1	4.79	6.42E-06
Ch25h	cholesterol 25-hydroxylase	4.77	3.34E-08
Sash3	SAM and SH3 domain containing 3	4.63	5.18E-08
Col3a1	collagen, type III, alpha 1	4.57	<2.2E-16
Apobr	apolipoprotein B receptor	4.51	1.51E-08
Tbxas1	thromboxane A synthase 1, platelet	4.48	4.66E-06
Mrc1	mannose receptor, C type 1	4.41	<2.2E-16
Hdc	histidine decarboxylase	4.38	2.12E-06
Emr1	EGF-like module containing, mucin-like, hormone receptor-like sequence 1	4.26	5.07E-10
Acta2	actin, alpha 2, smooth muscle, aorta	3.8	6.67E-06
Hmox1	heme oxygenase (decycling) 1	3.78	1.98E-06
Psap	prosaposin	3.52	5.18E-08
Fcgr2b	Fc receptor, IgG, low affinity IIb	3.46	6.42E-13
Cd44	CD44 antigen	3.45	6.42E-13
Lrrc33	leucine rich repeat containing 33	3.02	2.97E-06
Actg2	actin, gamma 2, smooth muscle, enteric	2.97	1.97E-06
Anxa1	annexin A1	2.93	2.99E-06
Lat	linker for activation of T cells	2.82	7.97E-11
Psmb8	proteasome (prosome, macropain) subunit, beta type 8 (large multifunctional peptidase 7)	2.81	5.79E-06
Trpv2	transient receptor potential cation channel, subfamily V, member 2	2.79	1.27E-10
Lcp1	lymphocyte cytosolic protein 1	2.79	2.63E-06
Mest	mesoderm specific transcript	2.69	8.08E-07
Man2b1	mannosidase 2, alpha B1	2.64	5.44E-07
BC013712	cDNA sequence BC013712	2.45	6.37E-06
Rab3il1	RAB3A interacting protein (rabin3)-like 1	2.42	1.97E-08
Rrm2	ribonucleotide reductase M2	2.39	1.57E-08
Mfap4	microfibrillar-associated protein 4	2.33	6.41E-06
Pfn1	profilin 1	2.31	2.43E-08
Cd163	CD163 antigen	2.3	5.12E-06
Tcirg1	T cell, immune regulator 1, ATPase, H+ transporting, lysosomal V0 protein A3	2.23	2.77E-06
Cst3	cystatin C	2.01	1.69E-06
Espl1	extra spindle poles-like 1 (S. cerevisiae)	1.95	1.98E-06
Frrs1	ferric-chelate reductase 1	1.95	2.23E-06
Tbc1d1	TBC1 domain family, member 1	1.88	6.41E-06
Bub1b	budding uninhibited by benzimidazoles 1 homolog, beta (S. cerevisiae)	1.86	6.55E-06
Gsta3	glutathione S-transferase, alpha 3	-4.85	5.14E-12
Fmo1	flavin containing monooxygenase 1	-3.79	7.54E-07
Tppp	tubulin polymerization promoting protein	-2.69	3.93E-10
Fxyd1	FXYD domain-containing ion transport regulator 1	-2.68	3.93E-10
1810020D17Rik	RIKEN cDNA 1810020D17 gene	-2.68	1.20E-09
Rorc	RAR-related orphan receptor gamma	-2.51	1.69E-06
Ppm1l	protein phosphatase 1 (formerly 2C)-like	-2.35	3.09E-08
Klf15	Kruppel-like factor 15	-2.34	1.73E-08
Amigo2	adhesion molecule with Ig like domain 2	-2.03	1.15E-06
Mcoln1	mucolipin 1	-1.93	3.59E-06
Kbtbd4	kelch repeat and BTB (POZ) domain containing 4	-1.91	2.87E-06

### Protein-protein interaction network

**Fig 5:**
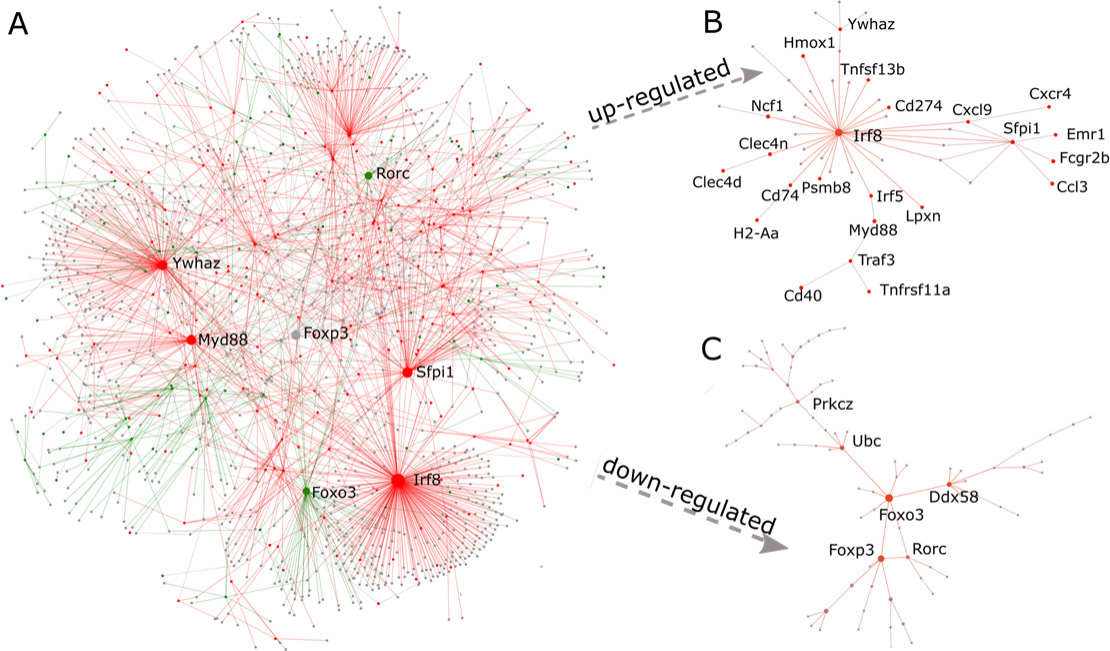
PPI subnetwork analysis of DE genes. (A) The subnetwork using all DE genes. Nodes in red indicate upregulated genes, nodes in green indicate down-regulated genes, and nodes in grey are interaction partners of the DE genes; (B) The PPI subnetwork for up-regulated genes; (C) The PPI subnetwork for down-regulated genes.

To understand the relationships among these co-regulated genes, we projected all DE genes into the mouse PPI network and obtained a densely connected subnetwork containing 253 of those DE genes and 1069 of their interacting partners (**Fig 5A**). For up-regulated genes, a total of 48 genes directly interact with each other and form a small subnetwork with *Irf8* at the center (**Fig 5B**). *Irf8* is a transcription factor that plays an important role in regulating lineage commitment and maturation of myeloid and lymphoid cells [73]. It is also associated with negative immunoregulatory functions such as inhibition of Th17 immune response [74]. This subnetwork also includes key immunoregulatory genes including *Cd274*, *Fcgr2b*, *Lpxn* and *Hmox1*. Cytokines and cytokine receptors include *Ccl3*, *Cxcl9 and Cxcr3*. It also contains *Myd88*, a key regulator of *Nfkb* transcription factor. For the down-regulated genes, we could not identify a direct interaction subnetwork. Instead, these genes require extra interactions partners to be connected (**Fig 5C**). The resulting subnetwork contains 51 genes with *Foxo3* at the central position. Interestingly, evidence of immunosuppression can be witnessed in this subnetwork. *Foxo3* is a transcription factor that can modulate the function of dendritic cells to control the magnitude of T cell immune responses [75]. It also interacts with transcription factor *Foxp3* to regulate Treg cell development. The subnetwork also contains several other immune genes such as *Rorc*, a transcription factor for Th17 cells, and *Strap*, a negative regulator of Tgf-beta signaling.

## Discussion

Host responses to helminth infections are a well-studied subject, however, to the best of our knowledge, no comprehensive gene expression meta-analysis has been reported in this area. In this paper, we reported the results from a comprehensive meta-analysis of host immune responses in helminthiases. In particular, by integrating datasets from multiple studies across different tissue types and helminth species, we were able to identify a robust and consistent pattern of gene expression, representing the core immune signature of host response during helminth infection. Functional enrichment analyses of these genes revealed that immune-related genes are up regulated and those primarily involved in metabolic processes are mainly down regulated. Up-regulated genes are mainly involved in inflammatory responses, immune regulation and Th2 immunity. These genes and functions will serve as a basis for elucidating immune pathways targeted by helminths and facilitate future mechanistic studies to characterize the interactions between host and helminth parasites at molecular level.

The hygiene hypothesis suggests that a lack of exposure to infectious agents such as parasitic helminths in early childhood can lead to an increased risk of developing chronic inflammatory, autoimmune and allergic diseases. Helminth infections, along with other microorganisms, are thought to play an important role in the proper functioning of immunoregulatory mechanisms [76]. Excessive immune reactivity in Th1, Th2 and Th17 responses is harmful [77]. Helminth infections have been associated with reduced responses to specific parasite antigens [78, 79] and more generally, to bystander antigens [80]. Our study shows a broad range of DE genes associated with immune responses, with a significant portion involved in modulation of immune functions. We observed a general up-regulation of genes involved in negative regulation of antigen receptor-mediated signaling. This set of genes consists of *Lpxn*, *Thy1*, *Lgals3* and *Ptpn6*. Lgals3 interacts with helminth glycans [81] and plays an important role in modulation of host responses during helminth infections [82]. The common up-regulation of these inhibitory genes suggests that they might be mediators of the immunomodulatory consequences of helminth infection. Additionally, implications of helminth infections in the attenuation of immune response associated with autoimmune diseases can be witnessed in our results as well. Indeed, we observed an up-regulation of genes involved in the PD-1 pathway and down-regulation of *Rorc*, a key transcription factor involved in Th17 development. Similarly, we observed an up-regulation of transcription factor *Irf8*, which has inhibitory activity towards the differentiation of Th17 cell [74]. It is possible that helminth infection reduces incidence of autoimmune disorders through negative regulation of Th17 response.

The induction of Th2-type immune responses is mediated by multiple factors. Tissue injuries caused by parasite invasion can promote a Th2-type response for wound repair. Recent studies have shown that inflammatory responses associated with a sterile wound induced recruitment of AAM to the site of injury; depletion of these cells resulted in delayed wound repair [83]. However, an injury alone is not sufficient to maintain a Th2-type response; an adaptive response involving Th2 CD4^+^ T cells is required [84]. We observed up-regulation of AAM markers in addition to a set of genes involved in hemostasis, tissue repair and extracellular matrix remodelling. These changes in gene expression may be associated with responses to tissue injury caused by parasite migration or tissue invasion within the host. Helminths themselves can also promote Th2 responses by producing excretory-secretory products and expressing Th2-inducing surface molecules. Many helminth glycans can mediate immunoregulation [85]. *Clec10a*, a C-type lectin receptor, recognizes *LacdiNac GalNacB1-4GlcNac* (LDN) and *GalNacA1-O-Thr/Ser* (Tn), glycan structures which are present in multiple helminth species, including *S*. *mansoni* and *T*. *spiralis* [85]. In the case of *S*. *mansoni*, *Clec10a*, along with *CD209* and *Mrc1*, recognizes soluble egg antigens and mediates Th2 responses through MHC class II antigen presentation [86]. We found both *Mrc1* and *Clec10a* to be consistently up-regulated by helminth infections. Similarly, the beta-galactose-recognizing family of lectins, also known as galectins (*Lgals3*), may interact with helminth glycans, as many of them contain terminal galactose [87]. Specifically, galectin-3 has been shown to interact with LDN. In a murine *in vivo* study, LDN antigens induced formation of Th2-associated granulomas in liver, possibly mediated by interaction with *Lgals3* due to its high up-regulation in granulomas [88]. In our data, a common up-regulation of these genes confirms the results of previous studies and indicates that these gene products may be involved in multiple host-parasite interactions.

There are several limitations associated with this current meta-analysis. The first limitation is the relatively small number of datasets (containing nine datasets and 55 samples in total) used in this study, despite our best efforts in data collection, processing and integration using advanced statistics and visualization techniques; Secondly, the results are based on murine models which may not be able to accurately reflect the immune responses that occur in human clinical settings as shown in a recent study on chronic clinical hepatic schistosomiasis [89]. Thirdly, the results could potentially bias towards liver immune responses as four out of the nine datasets were from the liver tissue. Despite these limitations, our analyses reveal a general host immune signature to helminth infections, with constituent genes and biological processes to enable better understanding of host-parasites interactions. The signature will be further refined and validated with increasing number of gene expression studies in the future.

## Supporting Information

S1 Table The complete list of 39 datasets together with their Accession numbers, brief descriptions and associated references if available. (DOCX)

S2 Table The 691 differentially expressed genes identified from our meta-analysis using adjusted p value cut-off of 0.01. (CSV)

S3 Table The differential analysis result for all genes ranked by their adjusted p values. (CSV)

S1 Fig PCA plot before batch effect correction. All samples are represented by different symbols with shapes according to different studies and colors based on their experimental conditions.
(TIFF)

S2 Fig PCA plot after batch effect correction. All samples are represented by different symbols with shapes according to different studies and colors based on their experimental conditions.
(TIFF)

S3 Fig PCA plot after batch effect correction. All samples are represented by different symbols with shapes according to experimental conditions and colors based on their strains.
(TIFF)

S4 Fig Scatter plot showing expression values of two genes before batch effect correction. The symbols are shaped according to their experimental conditions with colors based on their dataset memberships.
(TIFF)

S5 Fig Scatter plot showing expression values of two genes after batch effect correction. The symbols are shaped according to their experimental conditions with colors based on their dataset memberships.
(TIFF)
