# Supplementary material for: Comprehensive Transcriptome Meta-analysis to Characterize Host Immune Responses in Helminth Infections
Source: PLoS Negl Trop Dis. 2016 Apr 8;10(4):e0004624. doi: 10.1371/journal.pntd.0004624 (PMC4826001; doi:10.1371/journal.pntd.0004624)
Supplement: S1 Table — (DOCX) [file pntd.0004624.s001.docx]

| **Access number** | **Study** | **Reference** |
| --- | --- | --- |
| E-GEOD-59276 | Characterising Host Gene Expression during the Recovery from Hepatic Schistosomiasis Japonica | Unpublished |
| E-ERAD-59 | Transcriptomics of the immune response to Schistosomiasis | Unpublished |
| E-GEOD-1382 | Transcription profiling of mouse IL10-/- dendritic cells pulsed for 6h with 0, SEA, LPS, or co-pulsed with SEA/LPS together to compare changes in LPS-induced gene expression mediated by SEA (Schistosome soluble egg antigen) | Kane, Colleen M., et al. "Helminth antigens modulate TLR-initiated dendritic cell activation." *The Journal of Immunology* 173.12 (2004): 7454-7461. |
| E-GEOD-15454 | Profiling of filarial induced genes in lymphatic endothelial cells | Unpublished |
| E-GEOD-2135 | Transcription profiling of human filaria induced monocyte dysfunction and its reversal following treatment | Semnani, Roshanak Tolouei, et al. "Filaria-induced monocyte dysfunction and its reversal following treatment." *Infection and immunity* 74.8 (2006): 4409-4417. |
| E-GEOD-25275 | Gene expression patterns of biopsies from a colonoscopy taken in 2007 of an ulcerative colitis patient infected with Trichuris trichiura | Unpublished |
| E-GEOD-25276 | Gene expression patterns of biopsies from a colonoscopy taken in 2008 of an ulcerative colitis patient infected with Trichuris trichiura | Unpublished |
| E-GEOD-25277 | Gene expression patterns of biopsies from a colonoscopy taken in 2009 of an ulcerative colitis patient infected with Trichuris trichiura | Unpublished |
| E-GEOD-25713 | Differential Expression of Chemokine and Matrix Re-Modelling Genes Explains Contrasting Schistosoma japonicum-induced Hepatopathology in Murine Models | Perry, Carly R., et al. "Differential expression of chemokine and matrix re-modelling genes is associated with contrasting schistosome-induced hepatopathology in murine models." *PLoS Negl Trop Dis* 5.6 (2011): e1178. |
| E-GEOD-31265 | Gene expression changes in murine ileum during inflammation: Controls vs. intestinal schistosomiasis and TNBS-ileitis | Avula, Leela Rani, et al. "Whole-genome microarray analysis and functional characterization reveal distinct gene expression profiles and patterns in two mouse models of ileal inflammation." *BMC genomics* 13.1 (2012): 1. |
| E-GEOD-31894 | Regulation of global gene expression in human Loa loa infection is a function of chronicity | Steel, Cathy, Sudhir Varma, and Thomas B. Nutman. "Regulation of global gene expression in human Loa loa infection is a function of chronicity." *PLoS Negl Trop Dis* 6.2 (2012): e1527. |
| E-GEOD-3414 | Transcription profiling of lungs from SCID and wild type mice infected with L3 stage infectious Nippostrongylus brasiliensis to examine the innate immune response to helminth infection in the lung | Reece, Joshua J., Mark C. Siracusa, and Alan L. Scott. "Innate immune responses to lung-stage helminth infection induce alternatively activated alveolar macrophages." *Infection and immunity* 74.9 (2006): 4970-4981. |
| E-GEOD-5555 | Transcription profiling of mouse infected with hookworm to investigate induced persistent changes to the immunological environment of the lung | Reece, Joshua J., et al. "Hookworm-induced persistent changes to the immunological environment of the lung." *Infection and immunity* 76.8 (2008): 3511-3524. |
| E-GEOD-55941 | Genome-wide expression analysis of the effects of a parasitic nematode's secreted exosomes on mammalian cells | Buck, Amy H., et al. "Exosomes secreted by nematode parasites transfer small RNAs to mammalian cells and modulate innate immunity." *Nature communications* 5 (2014). |
| E-MEXP-3920 | Transcription profiling by array of mouse bone marrow derived macrophages stimulated with helminth larvae and immune serum | Esser-von Bieren, Julia, et al. "Antibodies trap tissue migrating helminth larvae and prevent tissue damage by driving IL-4Rα-independent alternative differentiation of macrophages." *PLoS Pathog* 9.11 (2013): e1003771. |
| E-MTAB-2100 | Transcription profiling by array of regulatory B-lymphocytes isolated from mice which were infected by male Schistosoma mansoni and uninfected controls | Amu, Sylvie, et al. "Regulatory B cells prevent and reverse allergic airway inflammation via FoxP3-positive T regulatory cells in a murine model." *Journal of Allergy and Clinical Immunology* 125.5 (2010): 1114-1124. |
| E-TABM-938 | Transcription profiling of human blood from patients with acute and chronic infection with the geohelminth ascaris lumbricoides | Ortiz, Miguel Reina, et al. "Effects of chronic ascariasis and trichuriasis on cytokine production and gene expression in human blood: a cross-sectional study." *PLoS Negl Trop Dis* 5.6 (2011): e1157. |
| GSE14367 | Temporal Expression of Chemokines Dictates the Hepatic Inflammatory Infiltrate in a Murine Model of Schistosomiasis. | Burke ML, McManus DP, Ramm GA, Duke M et al. Temporal expression of chemokines dictates the hepatic inflammatory infiltrate in a murine model of schistosomiasis. PLoS Negl Trop Dis 2010 Feb 9;4(2):e598. |
| GSE15447 | Gene expression profiling in mouse liver infected with Clonorchis sinensis metacercariae. | Kim DM, Ko BS, Ju JW, Cho SH et al. Gene expression profiling in mouse liver infected with Clonorchis sinensis metacercariae. Parasitol Res 2009 Dec;106(1):269-78. |
| GSE17544 | A novel and divergent role of granzyme A and B in resistance to helminth infection | Hartmann W, Marsland BJ, Otto B, Urny J et al. A novel and divergent role of granzyme A and B in resistance to helminth infection. J Immunol 2011 Feb 15;186(4):2472-81 |
| GSE17580 | Pronounced Phenotype in Activated Treg Cells during a Chronic Helminth Infection | Layland LE, Mages J, Loddenkemper C, Hoerauf A et al. Pronounced phenotype in activated regulatory T cells during a chronic helminth infection. J Immunol 2010 Jan 15;184(2):713-24. |
| GSE19525 | Gene expression in the Schistosoma japonicum infected spleen | Burke ML, McManus DP, Ramm GA, Duke M et al. Co-ordinated gene expression in the liver and spleen during Schistosoma japonicum infection regulates cell migration. PLoS Negl Trop Dis 2010 May 18;4(5):e686. |
| GSE24376 | Time course of gene expression profiling in the liver of experimental mice infected with Echinococcus multilocularis | Lin, Renyong, et al. "Time course of gene expression profiling in the liver of experimental mice infected with Echinococcus multilocularis." *PloS one* 6.1 (2011): e14557. |
| GSE27171 | Migrating Schistosoma japonicum schistosomula induce type-2 inflammation in the murine lung | Burke ML, McGarvey L, McSorley HJ, Bielefeldt-Ohmann H et al. Migrating Schistosoma japonicum schistosomula induce an innate immune response and wound healing in the murine lung. Mol Immunol 2011 Oct;49(1-2):191-200. |
| GSE360 | Macrophages and dendritic cells exposed to phylogenetically distinct parasites | Chaussabel D, Semnani RT, McDowell MA, Sacks D et al. Unique gene expression profiles of human macrophages and dendritic cells to phylogenetically distinct parasites. Blood 2003 Jul 15;102(2):672-81. |
| GSE38802 | MicroRNA expression profiles associated with anti-schistosome features in Microtus fortis | Han, Hongxiao, et al. "Differential expression of microRNAs in the non-permissive Schistosome Host Microtus fortis under Schistosome infection." *PloS one* 8.12 (2013): e85080. |
| GSE41941 | Neutrophils play a key role in schistosome-induced hepatic granuloma formation | Chuah C, Jones MK, Burke ML, Owen HC et al. Spatial and temporal transcriptomics of Schistosoma japonicum-induced hepatic granuloma formation reveals novel roles for neutrophils. J Leukoc Biol 2013 Aug;94(2):353-65. |
| GSE42694 | Quiescent innate response to infective filariae by human Langerhans’ cells suggests a strategy of immune evasion | Boyd A, Bennuru S, Wang Y, Sanprasert V et al. Quiescent innate response to infective filariae by human Langerhans cells suggests a strategy of immune evasion. Infect Immun 2013 May;81(5):1420-9 |
| GSE45985 | MicroRNA-gene expression network in murine liver during Schistosoma japonicum infection | Cai P, Piao X, Liu S, Hou N et al. MicroRNA-gene expression network in murine liver during Schistosoma japonicum infection. PLoS One 2013;8(6):e67037. |
| GSE48936 | Murine Schistosoma-Induced Pulmonary Hypertension: Microarray Data | Graham BB, Chabon J, Kumar R, Kolosionek E et al. Protective role of IL-6 in vascular remodeling in Schistosoma pulmonary hypertension. Am J Respir Cell Mol Biol 2013 Dec;49(6):951-9. |
| GSE58116 | Helminth Infection Reactivates Latent γ-herpesvirus Via Cytokine Competition at a Viral Promoter | Reese TA, Wakeman BS, Choi HS, Hufford MM et al. Helminth infection reactivates latent γ-herpesvirus via cytokine competition at a viral promoter. Science 2014 Aug 1;345(6196):573-7. |
| GSE60537 | Human resistin alters lung mRNA expression from helminth-infected lungs. | Jang JC, Chen G, Wang SH, Barnes MA et al. Macrophage-derived human resistin is induced in multiple helminth infections and promotes inflammatory monocytes and increased parasite burden. PLoS Pathog 2015 Jan;11(1):e1004579. PMID |
| GSE61376 | Chronic clinical hepatic schistosomiasis japonica | Gobert, Geoffrey N., et al. "Transcriptional profiling of chronic clinical hepatic schistosomiasis japonica indicates reduced metabolism and immune responses." *Parasitology* 142.12 (2015): 1453-1468. |
| GSE61460 | Splenic B cells from Hymenolepis diminuta-infected mice ameliorate colitis independent of T cells and via cooperation with macrophages | Reyes JL, Wang A, Fernando MR, Graepel R et al. Splenic B cells from Hymenolepis diminuta-infected mice ameliorate colitis independent of T cells and via cooperation with macrophages. J Immunol 2015 Jan 1;194(1):364-78. |
| GSE64071 | Ex vivo mouse model for hepatic schistosomiasis | Gobert, Geoffrey N., et al. "An Ex Vivo Model for Studying Hepatic Schistosomiasis and the Effect of Released Protein from Dying Eggs." *PLoS Negl Trop Dis* 9.5 (2015): e0003760. |
| GSE67136 | Eosinophil-derived IL-4 promotes nematode growth in an innate context | Huang, Lu, et al. "Eosinophils and IL-4 Support Nematode Growth Coincident with an Innate Response to Tissue Injury." *PLoS Pathog* 11.12 (2015): e1005347. |
| GSE67365 | Expression data from mouse intestinal epithelial cells infected with Trichinella spiralis | Unpublished |
| GSE69588 | Gene expression profile in the liver of BALB/c mice infected with Fasciola hepatica | Rojas-Caraballo J, López-Abán J, Fernández-Soto P, Vicente B et al. Gene Expression Profile in the Liver of BALB/c Mice Infected with Fasciola hepatica. PLoS One 2015;10(8):e0134910. |
| GSE69611 | Splenic gene expression profile of mice immunized with an anti-Fasciola hepatica vaccine candidate: insights into the immunological mechanisms leading to protection | Unpublished |
|  |  |  |
