# Supplementary figures and images for: Comprehensive Transcriptome Meta-analysis to Characterize Host Immune Responses in Helminth Infections
Source: PLoS Negl Trop Dis. 2016 Apr 8;10(4):e0004624. doi: 10.1371/journal.pntd.0004624 (PMC4826001; doi:10.1371/journal.pntd.0004624)

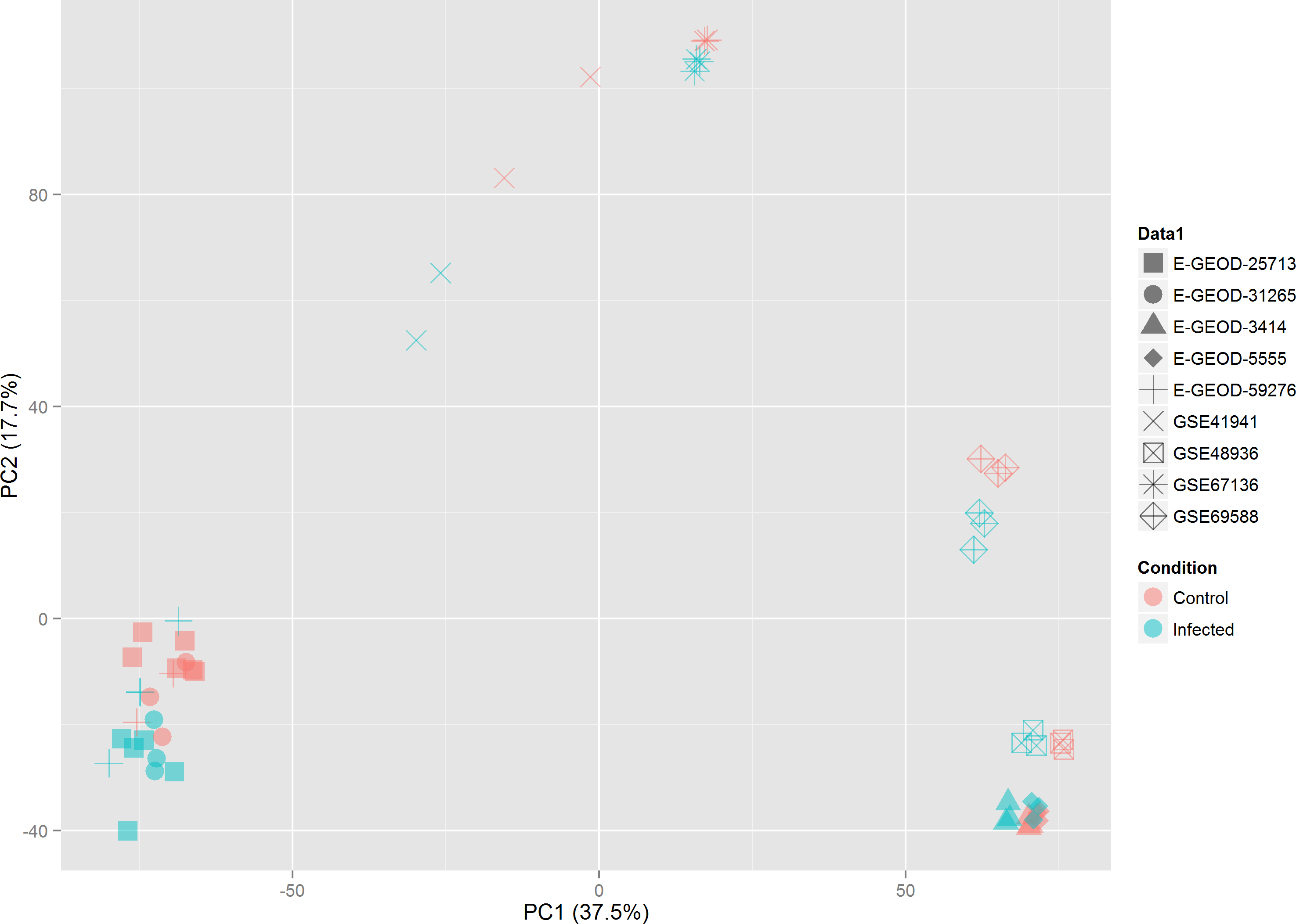

Supplement: S1 Fig — All samples are represented by different symbols with shapes according to different studies and colors based on their experimental conditions. (TIFF) [file pntd.0004624.s004.tiff]

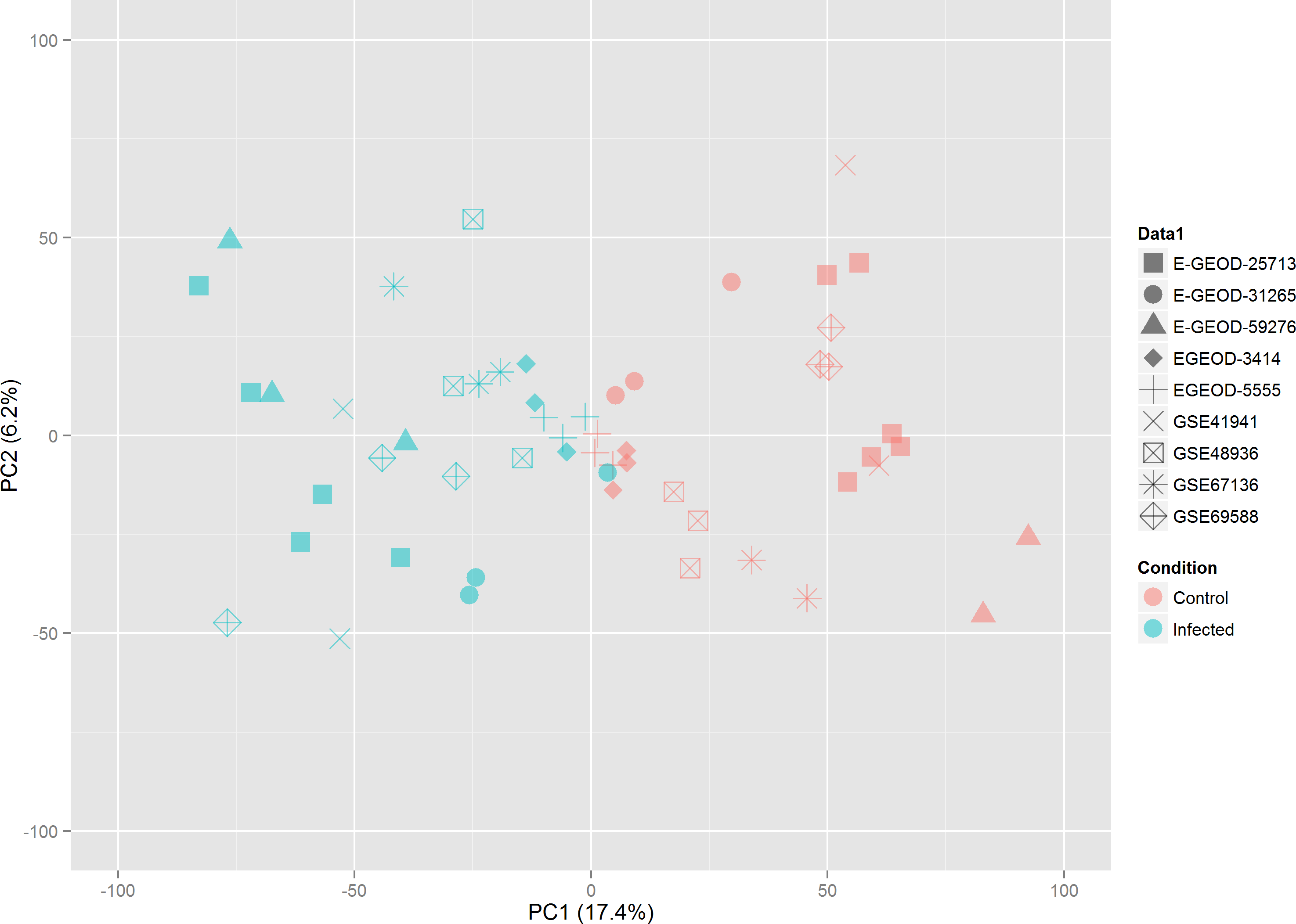

Supplement: S2 Fig — All samples are represented by different symbols with shapes according to different studies and colors based on their experimental conditions. (TIFF) [file pntd.0004624.s005.tiff]

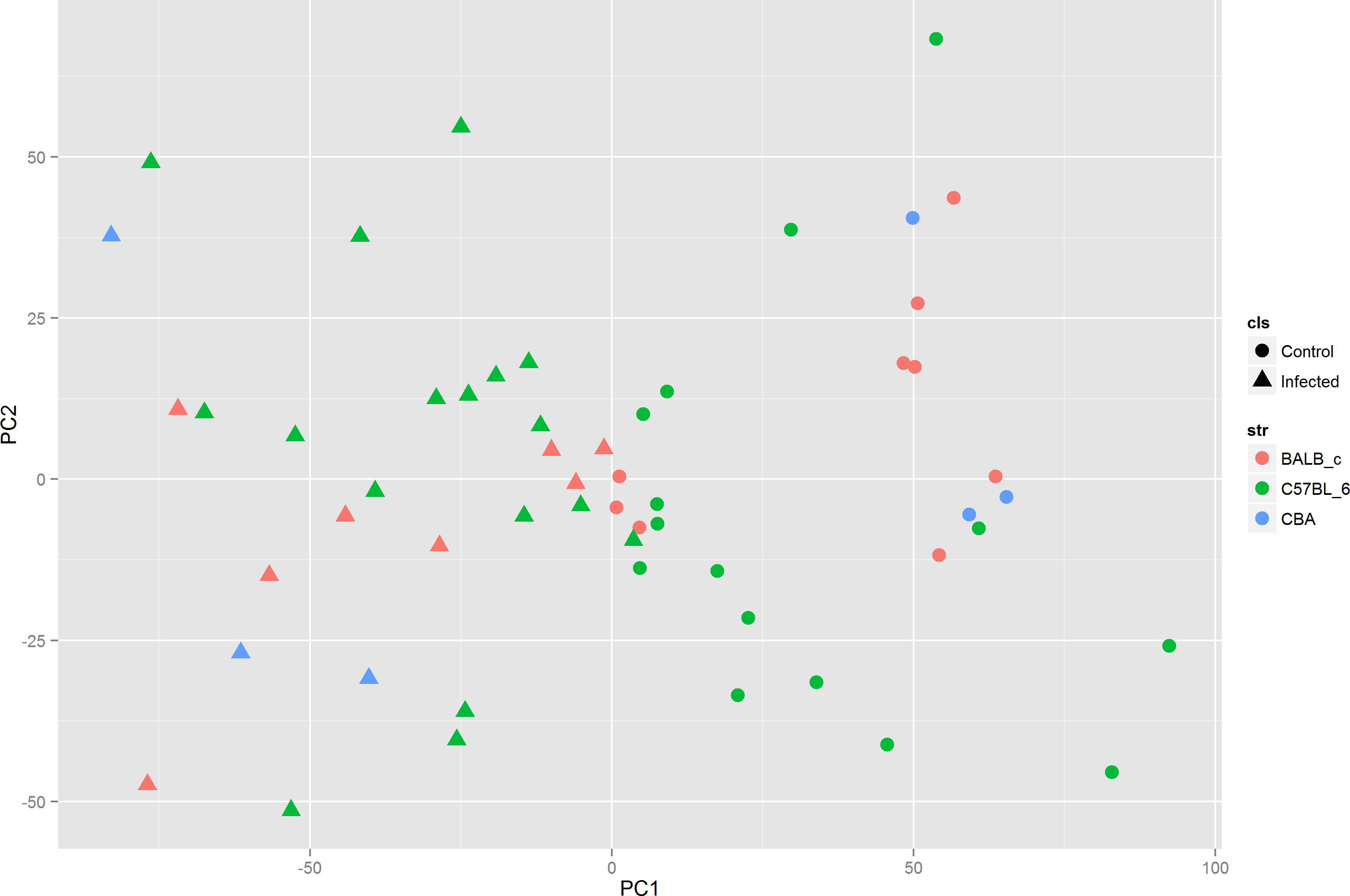

Supplement: S3 Fig — All samples are represented by different symbols with shapes according to experimental conditions and colors based on their strains. (TIFF) [file pntd.0004624.s006.tiff]

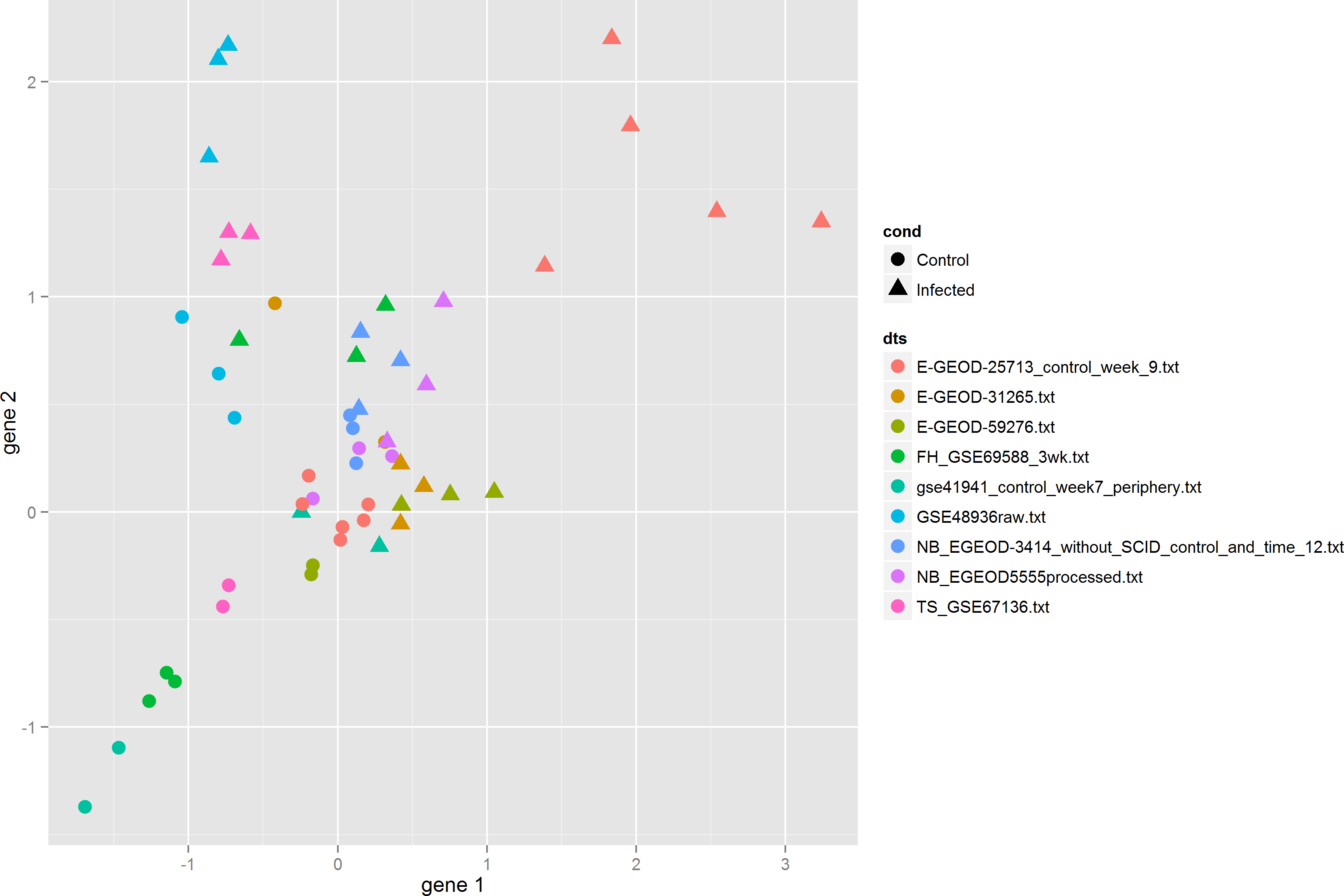

Supplement: S4 Fig — The symbols are shaped according to their experimental conditions with colors based on their dataset memberships. (TIFF) [file pntd.0004624.s007.tiff]

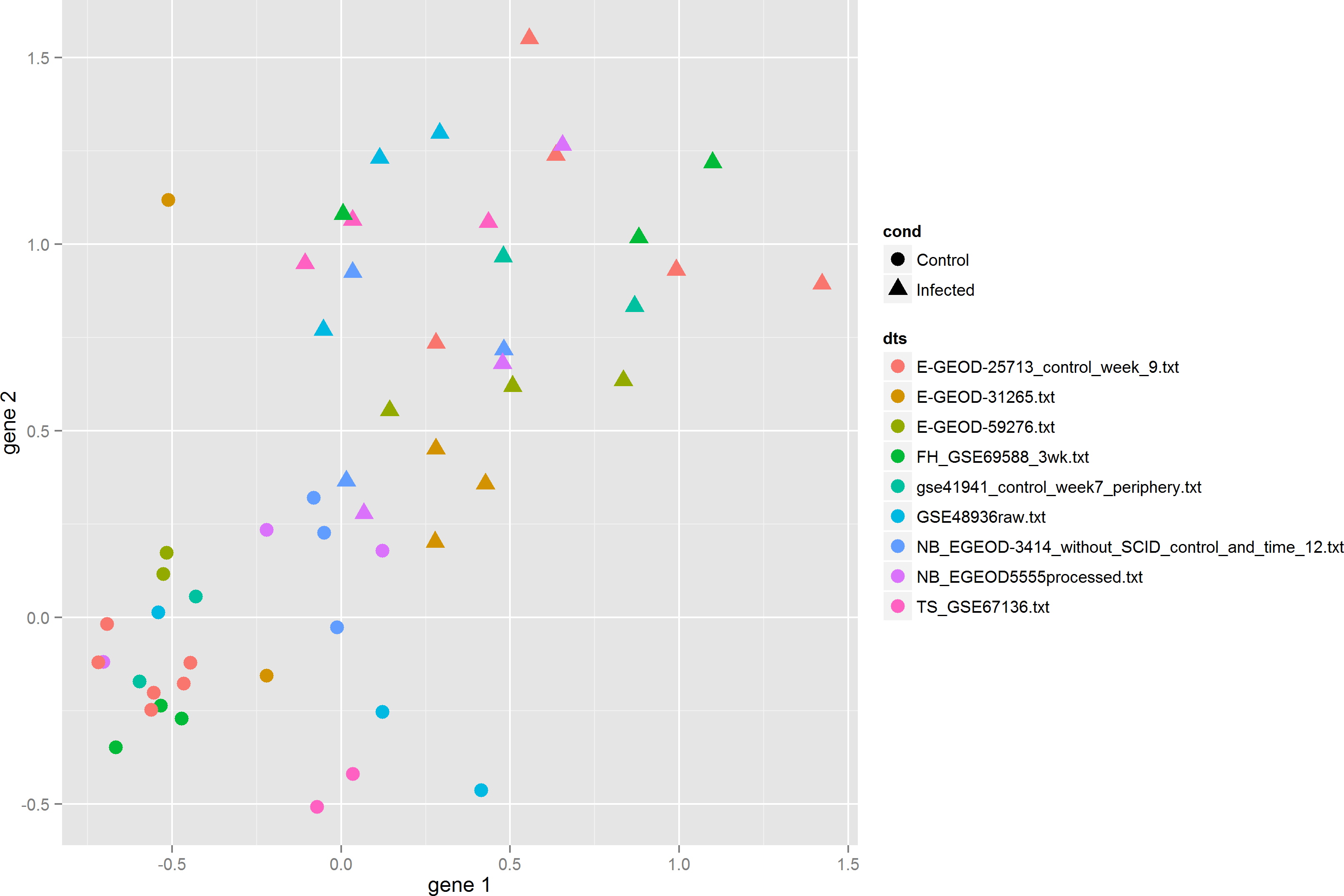

Supplement: S5 Fig — The symbols are shaped according to their experimental conditions with colors based on their dataset memberships. (TIFF) [file pntd.0004624.s008.tiff]
